# Dysfunctional Default Mode Network in Methadone Treated Patients Who Have a Higher Heroin Relapse Risk

**DOI:** 10.1038/srep15181

**Published:** 2015-10-15

**Authors:** Wei Li, Qiang Li, Defeng Wang, Wei Xiao, Kai Liu, Lin Shi, Jia Zhu, Yongbin Li, Xuejiao Yan, Jiajie Chen, Jianjun Ye, Zhe Li, Yarong Wang, Wei Wang

**Affiliations:** 1 Department of Radiology, Tangdu Hospital, The Fourth Military Medical University, Baqiao District, Xi’an, Shaanxi 710038, China; 2 Department of Imaging and Interventional Radiology, The Chinese University of Hong Kong, Shatin, N.T., Hong Kong; 3 Department of Medicine and Therapeutics, The Chinese University of Hong Kong, Shatin, N.T., Hong Kong

## Abstract

The purpose of this study was to identify whether heroin relapse is associated with changes in the functional connectivity of the default mode network (DMN) during methadone maintenance treatment (MMT). Resting-state functional magnetic resonance imaging (fMRI) data of chronic heroin relapsers (HR) (12 males, 1 female, age: 36.1 ± 6.9 years) and abstainers (HA) (11males, 2 female; age: 42.1 ± 8.1 years) were investigated with an independent component analysis to address the functional connectivity of their DMN. Group comparison was then performed between the relapsers and abstainers. Our study found that the left inferior temporal gyrus and the right superior occipital gyrus associated with DMN showed decreased functional connectivity in HR when compared with HA, while the left precuneus and the right middle cingulum had increased functional connectivity. Mean intensity signal, extracted from left inferior temporal gyrus of HR patients, showed a significant negative correlation corresponding to the degree of heroin relapse. These findings suggest that altered functional connectivity of DMN may contribute to the potential neurobiological mechanism(s) of heroin relapse and have a predictive value concerning heroin relapse under MMT.

Among current health and social problems, substance abuse remains one of the most prominent, with stimulant use ranked the costliest and most dangerous^1^. Repeated stimulant use leads to addiction, which is characterized by vulnerability to relapse^2^. Within South East Asia and particularly in China, heroin is the primary drug of abuse and it has become a serious social problem^3^. Examined data from literature has shown that patterns in heroin abuse follow the same relapse-rate curve as is exhibited in smoke and alcohol addiction with rates of post-treatment relapse as high as 50% within the space of one year^4^,^5^. Furthermore, large individual differences in the length of abstinence periods have been observed: With abstinence periods ranging from as little as one month to a year. This has prompted the need for an analysis of the neurobiology of relapsed heroin users in order to understand the factors that might be responsible for both the susceptibility to relapse and the duration of abstinence. This knowledge may aid in the development of novel therapies that will improve the effectiveness of current heroin rehabilitation interventions.

Previous studies have proved resting state functional connectivity (rs-FC) to be a useful tool in investigating brain networks through the analysis of synchronous low frequency blood oxygen level-dependent (BOLD) signal fluctuation^6^,^7^. Unlike the task-dependent methods of biomedical investigation, the use of rs-FC is particularly advantageous in that it does not require active patient participation. And yet, it enables the investigation of local neural responses and interregional connections for all brain regions within the same experimental protocol^8^. The rs-FC was targeted particularly towards regions which exhibit temporal correlated fluctuation in areas that are functionally related by the spatially distinct resting state networks (RSN). Independent Component Analysis (ICA) was used to identify the RSN in the resting state fMRI of heroin users. The protocol was closely matched to studies rs-FC studies previously performed in anorexia nervosa patients^8^. Because of rs-FC’s proven ability to expose aberrant neural connectivity, which underlies psychiatric disorders^9^, it was hypothesized that this protocol might be a poignant tool in studying the neuromorphology that is responsible for heroin drug relapse. While ICA data mining of fMRI data was used within prior drug studies^10^,^11^,^12^,^13^, this is the first time that a study of this sort has been done to analyze the RSN of heroin drug relapse.

The use of ICA analysis of rs-FC was used previously in heroin relapse studies that sought to identify possible alteration of functional connectivity of the DMN: A set of brain regions that exhibit robust low frequency oscillations during the resting state, but deactivate during activity^14^. These studies compared heroin users against non-addicted controls. They were able to elucidate an increased functional connectivity in the right hippocampus and a decreased functional connectivity in right dorsal anterior cingulate cortex and left caudate in the DMN of the controls^14^. A majority of the previous heroin fMRI studies on abnormal brain function in heroin-dependent individuals. One previous study focused on the resting-state abnormalities in heroin-dependent individuals and assessed the relationship between the resting-state functional connectivity changes and the duration of heroin use. The study showed that DMN of heroin-dependent individuals were changed compared with healthy subjects^15^. Another study suggested that drug addicts have an abnormal functional organization of the DMN. This was discussed as an addiction-related abnormally which increased memory processing but diminished cognitive control related to attention and self-monitoring (which may underlie the hypersensitivity toward drug related cues but a weakened strength of cognitive control in the state of addiction)^16^. All these findings underscore that the alteration of DMN is closely related to heroin use and consequent drug addiction. Thus, suggesting the pathological role of DMN in the process of drug addiction.

What current research efforts have not done, and what this study aims to do, is to compare the DMN of heroin relapse patients with that of heroin abstainers and to identify possible alterations which exist in the resting state functional connectivity of the DMN of a heroin relapser. The differences to be observed in the DMN between heroin relapsers and abstainers are supposed to give neurobiological explanation to the relapse condition, and can be referred to as evidence for predicting the treatment outcome. Basing on the findings of this study, an identification system, which can predict relapse, is proposed for heroin addiction patients. Accordingly, such a system would be helpful for better understanding the therapeutic effect of methadone treatment.

## Results

### Participants

Demographic information is presented in Table 1. No significant group difference was found between the two groups of HA and HR in terms of age, years of education, pre-baseline methadone dosage, follow-up daily methadone dosage, years of smoking, cigarettes smoking per day, years of heroin abuse, pre-baseline heroin abuse, depression and anxiety scores. Demographic information of the two groups matches which makes following analysis and group comparison results reliable.

### Independent Component Identification

**Figure 1:**
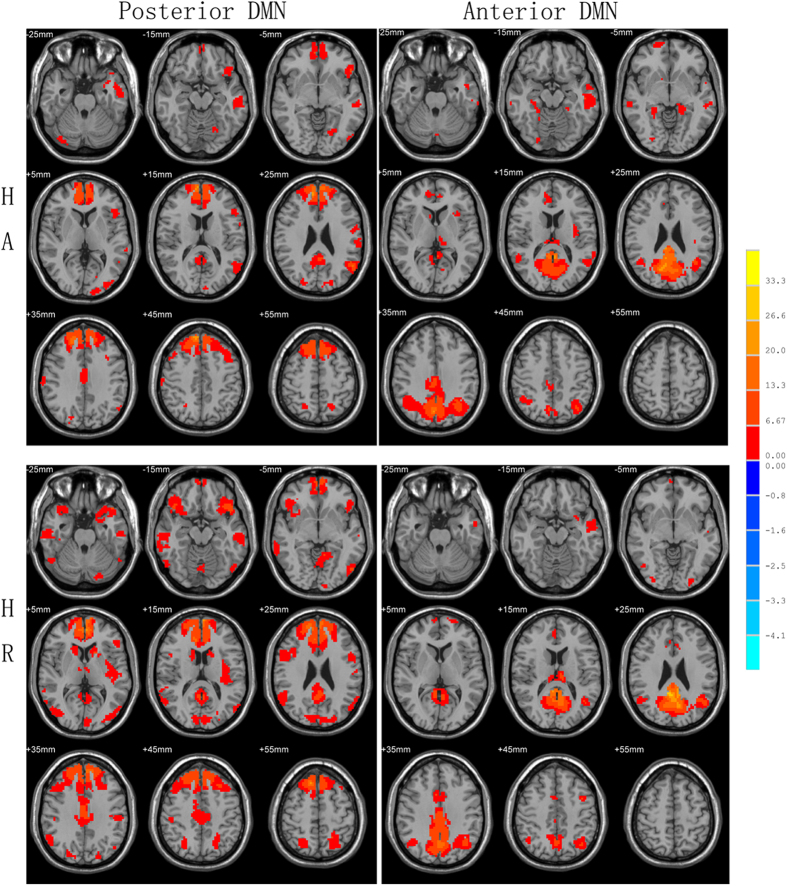
DMN one-sample results. DMN was divided into two parts of posterior and anterior DMN in ICA, the components’ thresholds were at a level of significance p < 0.05, FDR corrected with a minimal cluster size of 20.

Corresponding ICA images were independently selected from the two groups according to the templates presented by GIFT. One-sample t-test result of DMN from HA and HR are shown in Fig. 1. The DMN was separated into the posterior and anterior parts. The DMN components’ thresholds were at a level of significance p < 0.05, FDR corrected with a minimal cluster size of 20. As a result, regions selected in this study match well with classical DMN regions.

### Group Comparison

**Figure 2:**
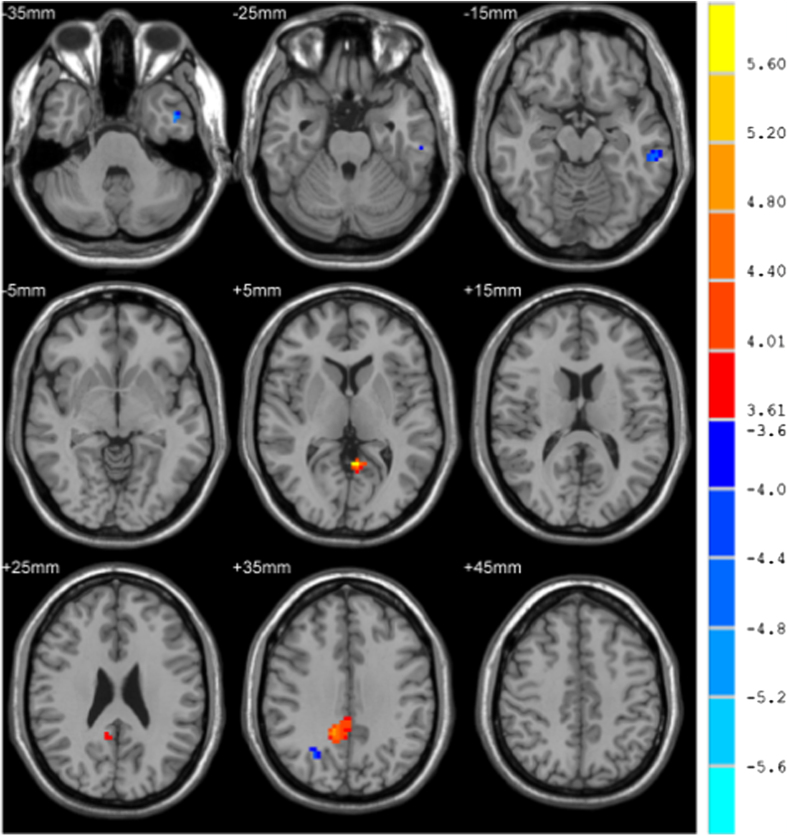
DMN two-sample result. The two sample t-test analysis took age and pre-baseline methadone dosage as covariates and the significance level was set at p < 0.05 (FDR corrected), minimal cluster size was set to 20 voxels.

We used age and methadone dosage as covariates for the group comparison and the difference in results is shown in Table 2 and Fig. 2. The left inferior temporal gyrus and right superior occipital gyrus associated with DMN showed a decrease in functional connectivity in the HR group compared with the HA group. The left precuneus and right middle cingulum show increased functional connectivity.

### Correlation results

**Figure 3:**
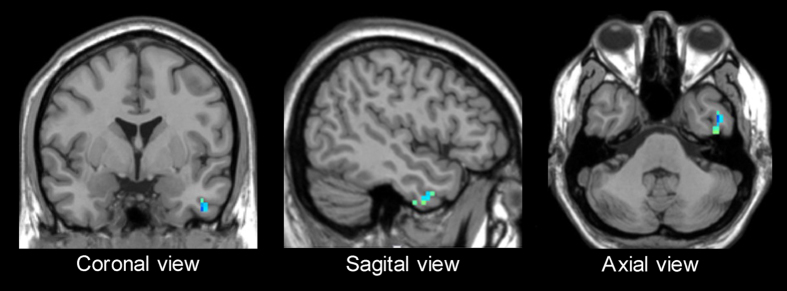
Function relevant cluster in DMN, Left inferior temporal gyrus; coordinates of local maxima [−45 0 −39]; T value at local maxima 5.2304; cluster size in voxels 23.

**Figure 4:**
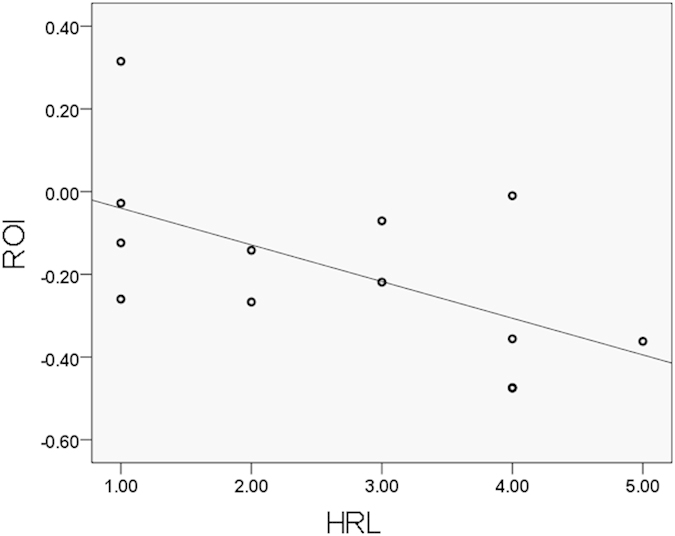
Correlation result of HRL and ROI-signal of HR (p = 0.038), significant negative correlation were found between HRL and ROI-signal (−0.578).

ROI signal abstracted from the left inferior temporal gyrus had a significant negative correlation with heroin relapse level( −0.578, p = 0.038), shown in Figs 3 and 4. HR with high relapse frequency has low z-value in the ROI.

## Discussion

The overarching goal of this study was to investigate the resting state functional connectivity in RSNs of patients who are susceptible to heroin relapse during MMT as compared to those who were able to sustain abstinence from heroin use. The main objective was to test the hypothesis that there exists significant relapse-related alteration of functional connectivity of the DMN between the two. Our study found that the left inferior temporal gyrus and right superior occipital gyrus were associated with a DMN decrease in functional connectivity for HR compared to HA. Additionally, the left precuneus and right middle cingulum show an increased functional connectivity in HR compared to HA. Intensity signals, extracted from the left inferior temporal gyrus of HR patients, showed a significant negative correlation with corresponding HRL.

Based on the results obtained, it was concluded that the clusters, which exhibited between-group differences, disproportionately drive DMN functional connectivity in heroin relapse patients.

As we hypothesized, significant differences in resting state functional connectivity were observed in the DMN of HR patients compared to HA. The DMN has proved pertinent to attention, self-monitoring and introspective thoughts and has a “sentinel” role within the resting state in which it enables the brain to broadly evaluate information from both external and internal environments^17^. Alterations in DMN have been shown to be responsible for the inability of patients with neuropsychiatric diseases to successfully undertake goal-directed activities, which require attentional orientation^17^. The changes in DMN observed in this study reveal an underlying a deficit in the inability of HR to successfully perform introspective mental processes. In addicted patients, the alteration of this internal awareness may contribute to the craving responses as a result of exposure to stress or drug cues. Craving is regarded as having an important role to play in the risk of relapse.

The left precuneus in DMN showed an increase connectivity in HR compared to HA in our study. The precuneus is implicated in memory retrieval, attentional tracking of stimuli^18^. It also plays an important role in cue reactivity, which could integrate information about smoking cues in environment that are processed in the extended visual system and relay that information to areas associated with motivational behavior and choice in the ACC PFC and striatum^19^. Based on existing research and how this relates to our study, HR with increasing functional connectivity in the precuneus could lead to a strong desire for heroin. Additionally, the alteration of the reactivity in the precuneus, which is related to heroin relapse, may develop into a promising strategy for future heroin addiction therapies, such as determining if the new strategy could reduce activity in the precuneus.

The cingulate region has been shown to play an important role in drug addiction. The literature comprehensively shows that its impairment remains as one of the most consistent findings in drug addiction disorders^20^,^21^,^22^,^23^,^24^,^25^,^26^. Disruption of cingulate activity in drug-addicted subjects could lead to impairments in self-monitoring, cognitive changes, and behavior control^27^,^28^. Our findings of increased activation in heroin relapse patients in the cingulate may be, in part, potentially related to these behavioral and cognitive impairments that arise due to heroin abuse. These findings also showed that heroin has wide impacts on the brains of heroin abusers. A plausible explanation for the high activity observed in relapse patients over abstainers are the changes in the cingulate region which are observed when a drug addict stops taking the drug.

Our results demonstrated a significant negative correlation between the signal intensity of left inferior temporal gyrus region and the HRL. This shows that heroin relapse reduces the resting state functional connectivity of the DMN by deactivating it as indicated in Fig 4. For this correlation, a Spearman’s correlation coefficient was observed. This finding is in line with the previous study which has suggested that longer duration of heroin use is related to the reduced metabolism of the inferior temporal cortex observed in PET imaging, and a higher risk of relapse^29^. On the other hand, the temporal cortex is considered to be a drug-specific region for heroin. Researchers showed that drug-dependent subjects had focal brain structural changes composed of significantly decreased gray matter densities in the temporal cortex relative to healthy comparison subjects^30^,^31^. The decrease in gray matter densities show the brain damage caused by drug addiction, which is connected with functional impairment of the temporal region. Varying degrees of decreased connectivity intensities of the temporal gyrus, which was found in this study, are quite likely markers indicating different degrees of brain damage. Severe drug addict patients who are suggested as high-possibility relapsers have weaker connectivity in the inferior temporal gyrus. Therefore, taken together, the functional connectivity alteration in the inferior temporal gyrus observed in the present study may be caused by former heroin use, with this impairment increasing the risk of heroin relapse.

In our study, the minimum description lengths (MDL) criterion was used to estimate the number of extracted components from the data. Basing on these estimates, 30 components were extracted, among which DMN was divided into posterior and anterior 2 components. Within DMN, some brain regions behave differently than others. Some previous resting-state fMRI study processed similar decomposition^32^.

There were some limitations to our study, which should be carefully considered in the interpretation of our findings. Firstly, due to the difficulty in collecting a large subject cohort with completed monthly follow-up data (especially for the subjects with a successful abstinence throughout 6 months) the sample size used in this study was inevitably small and could have limited the significance of the study. More importantly, the small subject number may affect the efficacy of ICA due to the dimensional unbalance between component numbers and sample size. Thus, an obvious way of improving future studies would be to increase the sample size. Secondly, we only used a 6-month follow-up result to differentiate between heroin abstainers and relapsers. Although we tried to control a series of confounding factors by matching them between groups and adding as nuisance covariates, there are still some factors which could not be fully covered in this study and may preclude a definitive conclusion. For example, the abstainers were six years older than the relapse patients, a fact that could allude to some correlation between age and drug relapse in general. More importantly, the duration between last heroin use and MR scan was not involved, and this may affect the calculation of the abstinence duration. Therefore, the conclusion as currently drawn should be interpreted with caution. Finally, the HRL that was used as an indicator of the likelihood of drug relapse is an arbitrary measure that is based on interviews with heroin abusers (and should therefore be treated as such). Future research should agree on an objective measurement for the quantification of HRL.

## Conclusion

In conclusion, this study demonstrated that heroin users with a risk of relapse possibly show different DMN functional connectivity patterns compared with abstainers, which may suggested a potential biomarker of heroin relapse. These findings suggest that altered functional connectivity of DMN may contribute to the potential neurobiology mechanisms of heroin relapse and perhaps have a predictive value for heroin relapse probability under MMT. Moreover, these results may be helpful to predict the outcome of addiction treatment and to understand its related therapeutic mechanism(s).

## Methods

The present study was approved by the Institutional Board of the Fourth Military Medical University, China, and conducted in accordance with the Declaration of Helsinki.

### Subjects

The participants were comprised of 13 heroin abstinent patients (HA; 12 males, 1 female; mean age 42.1 ± 8.1 years) and 13 heroin relapse patients (HR; 11 males, 2 female; mean age 36.1 ± 6.9 years). All the patients undergoing MMT were recruited from the Xi’an BaQiao Methadone Substitution Treatment Center. Primary inclusion criteria for the subjects were as follows: (1) met the DSM-IV (American Psychiatric Association) diagnostic criteria for heroin addiction; (2) came to the center to seek medical help of their own volition; (3) were under MMT and received stable treatment for at least three months. Participants of all study groups were excluded if they had any neuropsychiatric disorder including organic brain syndrome and schizophrenia. Other exclusion criteria for all subjects were: (1) had a current medical illness; (2) had a history of head trauma; (3) showed any contraindication during MRI examination. The comorbidity rate of anxiety and depression in drug addiction patients is high and the Beck depression Inventory (BDI) and Hamilton Anxiety Scale (HAMA) were performed respectively for the purpose of evaluating what, if any, influence these would have upon the resting state functional connectivity analysis.

All participants gave written informed consent before entering the study in accordance with the requirements of the Institutional Review Board of the Tangdu Hospital.

### Relapse Detection and Heroin Relapse Level

A six month’s follow-up occurred after fMRI scanning to determine the baseline (which was defined at the time of this MRI scan). For each of the subjects, a structured interview along with a urine test occurred monthly. Participants were considered as relapse patients if they had used heroin (urinalysis results shown positive or self-reported heroin use) at anytime during the six months. In this paper, in order to evaluate the severity of relapse in HR patients, heroin relapse level (HRL) was proposed based on the relapse frequency during the follow-up period.

### Data Acquisition

All MRI scans were performed at the same baseline levels in the Imaging Center of the Tangdu Hospital, the Fourth Military Medical University. Participants were instructed to lie still with their eyes opened, remain awake and not to think anything specific for the resting state session. A routine structure T2WI image was conducted to exclude any gross cerebral abnormality. All MR data were acquired with 3.0 T GE Signa Excite HD whole-body MRI system. The functional component of the image was acquired by using a spin-echo EPI (echo planar imaging) with following parameters: number of volumes 150, number of slices 32, repetition time 2000ms, echo time 30 ms, flip angle 90°, slice thickness 4mm with no gap, field-of-view 256*256 mm, matrix = 64*64. The whole scanning for each participant lasted for about 8 minutes. Foam pads were used for fixing the position of the head and reducing any artifacts due to movement.

### Data Preprocessing

Data Processing Assistant for Resting-State fMRI (DPARSF) within Matlab©® software was used in the data preprocessing of the functional images. The functional scans were realigned to the first image with the volume of 150, and head motion correction was processed to get the corresponding mean functional image. The images were normalized with the voxel size of 3 × 3 × 3 mm by using an EPI template(Montreal Neurological Institute brain template) and spatially smoothed with a FWHM of 8 mm.

### Independent component analysis (ICA)

The Group ICA of fMRI Toolbox (GIFT), as implemented in Matlab, was used to abstract independent components by recognizing the blood oxygen level-dependent (BOLD) signal for all participants. The number of components, being thirty in this study, was estimated using a minimum description length (MDL) criteria, modified to take into account spatial correlation. Corresponding ICA images were independently selected from the two groups according to the templates presented by GIFT. DMN was divided into two parts of posterior and anterior DMN, and between-group comparisons were performed respectively.

### Statistical analysis

The Resting State fMRI Data Analysis Toolkit (REST) was used for the statistical analysis. Corresponding individual subject components, representing the network of interest from the ICA back-reconstructed processes were entered into REST to facilitate group analysis. To test for differences in network homogeneity, individual-level network homogenous maps were entered using one-sample t-tests. For each selected component, a gray mask (threshold 0.2) was used to confine the one-sample test analysis with gray matter. A union mask of the significant voxels (p < 0.01, FDR corrected) of the one-sample t-test results from each group was made in order to limit between-group comparison. The two sample t-test analysis took age and pre-baseline methadone dosage as covariates and the significance level was set at p < 0.05 (FDR corrected), minimal cluster size was set to 20 voxels.

### ROI-signal analysis

To analyze the relationship between the brain network and HRL, the mean signal intensity of the regions of interest (ROI) was extracted for each HR. Partial correlation analysis were processed to analyze the correlation between ROI-signal and HRL taking age and pre-baseline methadone dosage as covariates. The demographic and correlation analysis was performed by the Statistical Package for the Social Sciences version 13.0 (SPSS Inc., Chicago, IL, USA) software.

## Additional Information

**How to cite this article**: Li, W. *et al.* Dysfunctional Default Mode Network in Methadone Treated Patients Who Have a Higher Heroin Relapse Risk. *Sci. Rep.* **5**, 15181; doi: 10.1038/srep15181 (2015).

## Figures and Tables

**Table 1:** Demographic Information.

Demographic Data	HA(n = 13)	HR(n = 13)	t	p
Age(year)	42.1 ± 8.1	36.1 ± 6.9	2.03	0.053
Years of education(year)	9.5 ± 2.2	9.7 ± 3.4	−0.21	0.839
Years of smoking(year)	17.9 ± 4.6	18.8 ± 4.3	−0.48	0.633
Cigarettes smoking per day	17.7 ± 8.6	16.4 ± 9.5	0.37	0.659
Pre-baseline heroin abuse(g)	767.6 ± 582.8	873.6 ± 1186.5	−0.29	0.180
Pre-baseline Methadone dosage(L)	49.0 ± 41.8	38.6 ± 22.9	0.79	0.056
Follow-up daily Methadone dosage(ml)	43.8 ± 18.8	48.6 ± 16.8	−0.69	0.442
BDI scores	11.2 ± 9.8	9.4 ± 7.9	0.51	0.617
HAMA scores	13.8 ± 9.7	12.8 ± 11.6	0.26	0.800

p value was obtained by two-sample t-test.

**Table 2:** DMN analysis results—between group differences.

Region(aal)^(31)^	Coordinates of local maxima	t value at local maxima	Cluster size in voxels
HR>HA			
Left precuneus	−3 −54 6	5.9558	22
Right middle cingulum	12 −54 42	5.6858	115
HA > HR			
Left inferior temporal gyrus	−45 0 −39	5.2304	23
Right superior occipital gyrus	0 −57 36	5.2227	26

Resulting statistical parametric maps are thresholded to p < 0.05 (FDR corrected);

the minimal cluster size was set to 20 voxels.
